# Combined maternal and postnatal high-fat diet leads to metabolic syndrome and is effectively reversed by resveratrol: a multiple-organ study

**DOI:** 10.1038/s41598-018-24010-0

**Published:** 2018-04-04

**Authors:** Jiunn-Ming Sheen, Hong-Ren Yu, You-Lin Tain, Wan-Long Tsai, Mao-Meng Tiao, I-Chun Lin, Ching-Chou Tsai, Yu-Ju Lin, Li-Tung Huang

**Affiliations:** 1 Department of Pediatrics, Kaohsiung Chang Gung Memorial Hospital and Chang Gung University College of Medicine, Kaohsiung, Taiwan; 2 Department of Medical Research, Kaohsiung Chang Gung Memorial Hospital, Kaohsiung, Taiwan; 3 Department of Obstetrics and Gynecology, Kaohsiung Chang Gung Memorial Hospital and Chang Gung University College of Medicine, Kaohsiung, Taiwan; 4 Department of Traditional Medicine, Chang Gung University, Linkow, Taiwan

## Abstract

This study aimed to study the impact of a combination of maternal and post-weaning high-fat diets and whether resveratrol was beneficial. Sprague-Dawley dams were fed either chow or a high-fat diet, before mating, during pregnancy, and into lactation. At weaning, their offspring were randomly fed chow or a high-fat diet. Four experimental groups were generated: CC (maternal/postnatal chow diet), HC (maternal high-fat/postnatal chow diet), CH (maternal chow/postnatal high-fat diet), and HH (maternal/postnatal high-fat diet). A fifth group consisted of HH plus resveratrol. The 4 month-old offspring of HH group had higher body weight, higher levels of plasma triglycerides, leptin, angiotensin I and angiotensin II and abnormal intraperitoneal glucose tolerance test results, which fulfilled the features of metabolic syndrome. The dysregulation of the renin-angiotensin system was seen in multiple organs. Sirtuin 1 expression/abundance was reduced by a maternal/postnatal high-fat diet, in all the organs examined. Resveratrol ameliorated most of the features of metabolic syndrome and molecular alterations. The administration of a high-fat diet in both periods showed interactive metabolic effects in the plasma and many organs. Our results suggest that a maternal high-fat diet sensitizes offspring to the adverse effects of subsequent high-fat intake on multiple organs.

## Introduction

Over 2.1 billion adults are estimated to be overweight or obese, at present, of whom 38% are women of childbearing age^1^. Maternal obesity/a high-fat diet may predispose offspring to altered energy balance, obesity, and metabolic syndrome^2–5^. Metabolic syndrome is a compilation of risk factors, including hypertension, dyslipidemia, obesity and insulin resistance. Metabolic syndrome is now considered a disease related to developmental disorder^6^. Previously conducted studies showed that a maternal high-fat diet, followed by a postnatal high-fat diet, increased the risk of metabolic syndrome^7,8^. However, the mechanistic link between mothers who are obese/on a high-fat diet and offspring with metabolic syndrome is not yet completely understood.

Sirtuin 1 (SIRT1) is a prototype mammalian NAD^(+)^-dependent protein deacetylase that has emerged as a key metabolic sensor in various metabolic tissues. Growing evidence suggests that SIRT1 regulates glucose and lipid metabolism through its deacetylase activity^9^. Moreover, accumulating evidence supports that obesity with chronic inflammation is associated with low levels of NAD^+^ and SIRT1^10^. SIRT1 might be a new therapeutic target for the prevention of diseases related to insulin resistance, such as metabolic syndrome and type 2 diabetes mellitus.

The renin-angiotensin system (RAS) contains many angiotensin peptides and can locally act in almost all the tissues of the body^11^. The RAS is closely associated with metabolic syndrome, and the inhibitors of the RAS have been shown to be able to prevent the onset of type 2 diabetes, in high-risk populations^12^. Previously conducted studies showed that SIRT1 and the RAS are mutually regulated in various cells/tissue^13–16^. Guberman *et al*. suggested that a maternal high-fat diet induced persistent alterations in the adipose RAS components of offspring, and this was further exacerbated by a postnatal high-fat diet^17^.

Resveratrol, a natural polyphenolic compound produced by plants in response to environmental stress, is found in red grape skin, peanuts, a variety of berries and medical plants, and has gained special interest as a calorie-restriction mimetic, based on data from rodents. The biological role of resveratrol is to initiate the activation of SIRT1, which epigenetically modifies and inactivates the acetylation of inflammatory proteins. When rodents were fed a high-fat diet, resveratrol treatment improved glucose homeostasis, mitochondrial function, lipid parameters, body weight, and survival^18^.

In the current study, we aimed to study the long-term effects of a combined maternal and postnatal high-fat diet on multiple organs, including adipose tissue, the pancreas, and the dorsal hippocampus. Particularly, we evaluated SIRT1, the RAS, and the effects of resveratrol.

## Results

### Body weight, calorie intake and biochemistry

Female rats on a high-fat diet were heavier than those in the control group since 1 week after taking a different diet (Supplemental Fig. 1A). Mating was arranged after 5 weeks on a different diet. At this time, female rats taking a high-fat diet had higher plasma aspartate aminotransferase (AST), alanine transaminase (ALT) and leptin levels (Table 1). There was no significant difference in the plasma total cholesterol (Chol), high-density lipoprotein (HDL), glucose, or triglyceride (TG) levels (Table 1).

**Table 1:** Weights, biochemical values of dams before mating.

	Control (n = 12)	HF (n = 13)
Body weight (g)	256.3 ± 3.1	291.3 ± 4.1*
Plasma AST (U/L)	83.6 ± 4.1	139.0 ± 12.8*
Plasma ALT (U/L)	37.3 ± 5.6	94.7 ± 8.5*
Plasma total Chol (mg/dL)	69.1 ± 7.1	86.8 ± 8.7
Plasma TG (mg/dL)	50.9 ± 8.7	68.8 ± 9.5
Plasma HDL (mg/dL)	50.6 ± 3.8	48.2 ± 3.8
Blood sugar (mg/dL)	218.6 ± 7.6	247.6 ± 12.6
Leptin (pg/mL)	1.21 ± 0.19	8.70 ± 1.14*

Values are the means ± S.E.M. AST, aspartate transaminase; ALT, alanine aminotransferase; Chol, cholesterol; TG, triglyceride; HDL, high-density lipoprotein. *Significantly different at *P* < 0.05 by Mann-Whitney U test.

The body weights of 2-day-old male offspring were lower in the group born to mothers taking a high-fat diet (Supplemental Fig. 1B). The offspring were weaned at 0.75 month of age, and were assigned to either the chow diet or high-fat diet group, from weaning until 4 months of age., We found that the body weight of the 2-month-old offspring was affected by postnatal high-fat diet. At 4 months of age, we found that the body weight and the plasma leptin level of the offspring were affected by both a maternal high-fat diet and postnatal high-fat diet. In addition, an interaction between maternal high-fat and postnatal high-fat diet, in terms of body weight and plasma leptin levels, was observed *Post hoc* analysis showed higher body weight and plasma leptin levels in the HH group than the CH group. These data indicated that maternal obesity/a high-fat diet led to increased body weight and plasma leptin levels, and sensitized offspring to a second hit, i.e., a postnatal high-fat diet. A Mann-Whitney U test showed that resveratrol treatment significantly reduced the plasma leptin level and body weight in the HH group. In regards to calorie intake per day from 2 to 4 months of age, a postnatal high-fat diet effect was observed; however, no effect of maternal high-fat diet was noted. Two-way analysis of variance (ANOVA) showed no significant interaction about calorie intake per day between maternal high-fat diet and postnatal high-fat diet. Mann-Whitney U test showed that rats in the resveratrol treatment group had lower daily calorie intake then the HH group. The plasma total Chol and TG levels were affected by both a maternal and postnatal high-fat diet. Resveratrol reversed the increased total Chol and TG levels in the HH group. In terms of plasma AST, ALT and HDL levels, a postnatal high-fat diet treatment effect was observed. Mann-Whitney U test showed that resveratrol treatment could reverse the increased plasma ALT and HDL levels in the HH group (Table 2).

**Table 2:** Weights, calorie intake, biochemical values and blood pressures (BPs) in male offspring.

	CC	HC	CH	HH	HHR
	(n = 13)	(n = 14)	(n = 14)	(n = 14)	(n = 14)
**Body weight** ^**@**^ **(g)**					
2 d/o	6.4 ± 0.3	5.9 ± 0.4	6.5 ± 0.3	6.0 ± 0.3	6.1 ± 0.2
0.75 m/o	50.3 ± 1.8	52.1 ± 1.3	51.5 ± 2.2	56.2 ± 3.2	51.3 ± 1.8
2 m/o	232.4 ± 2.7	230.7 ± 2.9	348.7 ± 8.5^**^	365.2 ± 14.0^**^	330.9 ± 16.9
4 m/o	387.2 ± 3.8	402.4 ± 6.3^!!^	611.0 ± 14.7^**^	721.7 ± 10.4^!!,**^	550.1 ± 17.8^#^
**Calorie intake (kcal/day)**					
0.75–2 m/o	30.5 ± 0.9	32.4 ± 1.0	28.9 ± 0.8	30.2 ± 0.7	28.5 ± 1.4
2–4 m/o	76.3 ± 0.9	75.1 ± 0.6	104.2 ± 2.5^**^	111.1 ± 1.8^**^	100.3 ± 1.0^#^
Plasma AST (U/L)	106.7 ± 6.4	105.4 ± 5.9	239.6 ± 17.9^**^	239.5 ± 23.4^**^	188.6 ± 16.2
Plasma ALT (U/L)	32 ± 0.9	34.8 ± 1.7	141.2 ± 14.7^**^	121.7 ± 12.4^**^	99.4 ± 11.8^#^
Plasma total Chol	50.1 ± 2.2	53.8 ± 2.5^!^	58.1 ± 2.2^**^	65.3 ± 3.5^!,**^	48.8 ± 2.1^##^
**(mg/dL)**					
Plasma TG (mg/dL)	89.1 ± 6	98.9 ± 9.8	77.1 ± 8.9^**^	105.7 ± 13.1^!,**^	62.9 ± 4.8^##^
Plasma HDL (mg/dL)	32.0 ± 2.2	30.8 ± 1.9	38.6 ± 1.8^**^	43.9 ± 2.4^**^	31.0 ± 1.7^##^
Leptin^@^ (pg/mL)	4.41 ± 0.38	5.87 ± 0.62^!!^	19.67 ± 1.58^**^	28.66 ± 1.44^!!,**^	21.56 ± 1.95^##^
Systolic BP (mmHg)	137.6 ± 6.6	152.1 ± 3.7	179.3 ± 8.3^**^	173.0 ± 4.7^**^	142.0 ± 4.5^#^
Diastolic BP (mmHg)	65.1 ± 5.6	86.5 ± 13.9	94.1 ± 7.4^*^	99.6 ± 9.2^*^	68.4 ± 4.2^#^
Mean BP (mmHg)	89.2 ± 1.7	108.3 ± 8.1	122.5 ± 7^**^	124.1 ± 5.8^**^	92.9 ± 3.2^#^

Values are the means ± S.E.M. Biochemical values and BPs were measured at 4 months of age. CC, offspring from maternal control diet with post-weaning control diet; HC offspring from maternal high-diet and postweaning control diet; CH offspring from maternal control diet and post-weaning high-fat diet, HH offspring from maternal high-fat diet and post-weaning high fat; d/o, day-old; m/o, month-old. The first four groups were analyzed by two-way ANOVA, followed by LSD *post hoc* tests. The therapeutic effect of resveratrol was evaluated by Mann-Whitney U test between HH and HHR group !,!! due to maternal high fat diet effect at *P* < 0.05 or < 0.01, respectively. *, ** due to postnatal high fat diet effect at *P* < 0.05 or <0.01, respectively. #, ## vs. HH group at *P* < 0.05 or <0.01, respectively. @ interaction present between maternal high fat and postnatal high fat diet.

### Blood pressure

The systolic, diastolic and mean BP increased through the consumption of a postnatal high-fat diet but were not affected by a maternal high-fat diet. Two-way ANOVA showed no significant interaction between maternal obesity/a high-fat diet and a postnatal high-fat diet. Resveratrol could reverse the increased systolic, diastolic and mean BP in the HH group (Table 2).

### Intraperitoneally injected glucose tolerance test (IPGTT)

**Figure 1:**
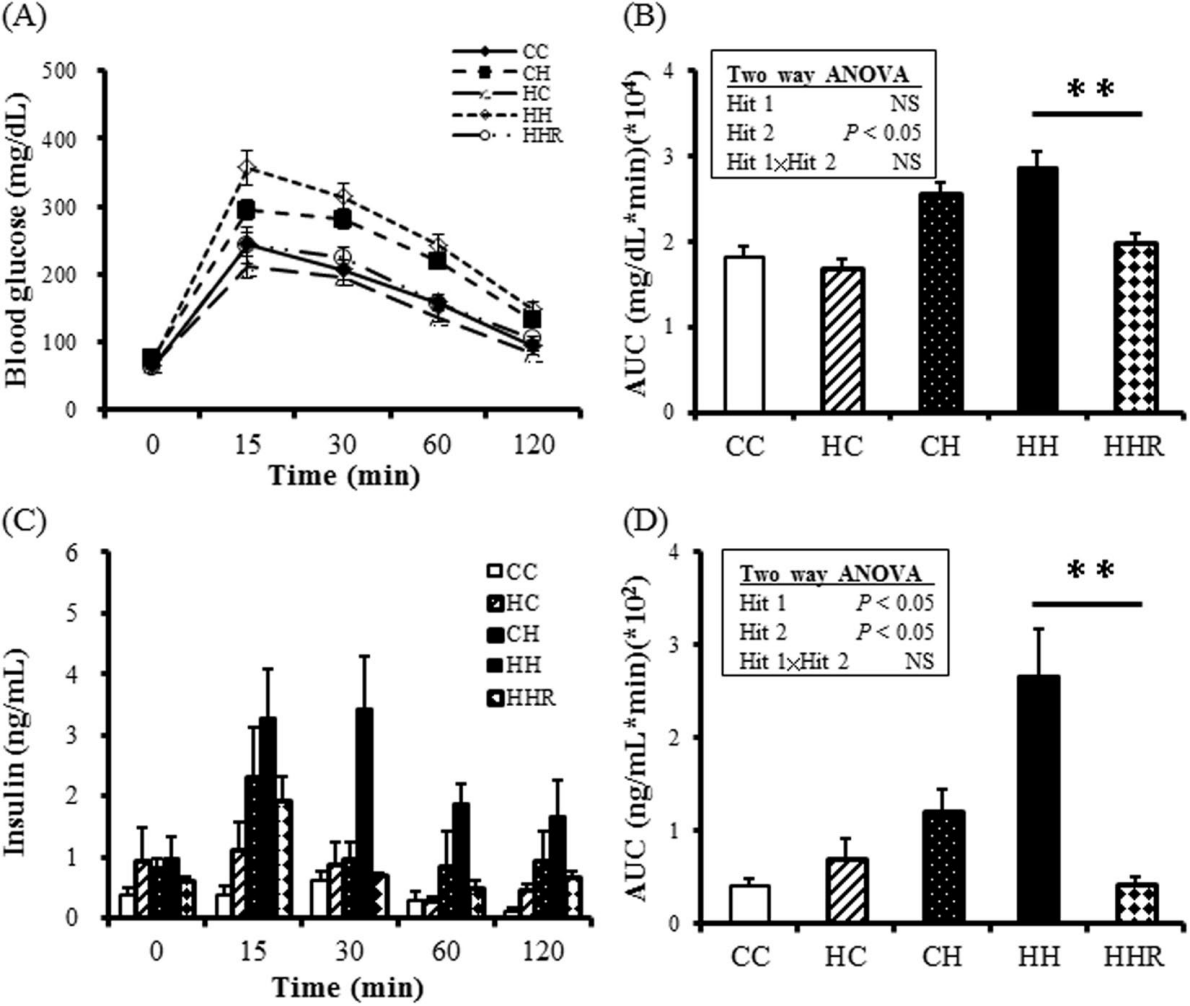
Intraperitoneal glucose tolerance test (IPGTT). (**A**) Serum glucose kinetics (**B**) Area under glucose curve (**C**) Serum insulin kinetics (**D**) Area under insulin curve during the IPGTT. Five experimental groups (n = 13–14 per group): CC: maternal rat chow diet + postnatal chow diet; HC: maternal obesity/high-fat diet + postnatal chow diet; CH: maternal rat chow diet + postnatal high-fat diet; HH: maternal obesity/high-fat diet + postnatal high-fat diet; HHR: maternal obesity/high-fat diet + postnatal high-fat diet + resveratrol. Hit 1, maternal high-fat diet; Hit 2, postnatal high-fat diet. Two-way ANOVA was used to assess the statistical significance of differences among groups and the therapeutic effect of resveratrol was evaluated by Mann-Whitney U test. **P < 0.01.

In terms of glucose AUC, two-way ANOVA showed no effect of maternal high-fat diet but a significant effect on postnatal high-fat diet. Resveratrol could reverse the sugar AUC value in the HH group. In terms of insulin AUC, two-way ANOVA showed both significant effects of maternal high-fat diet and postnatal high-fat diet. Resveratrol could reverse the insulin AUC value in the HH group (Fig. 1).

### Plasma angiotensin I and angiotensin II

**Figure 2:**
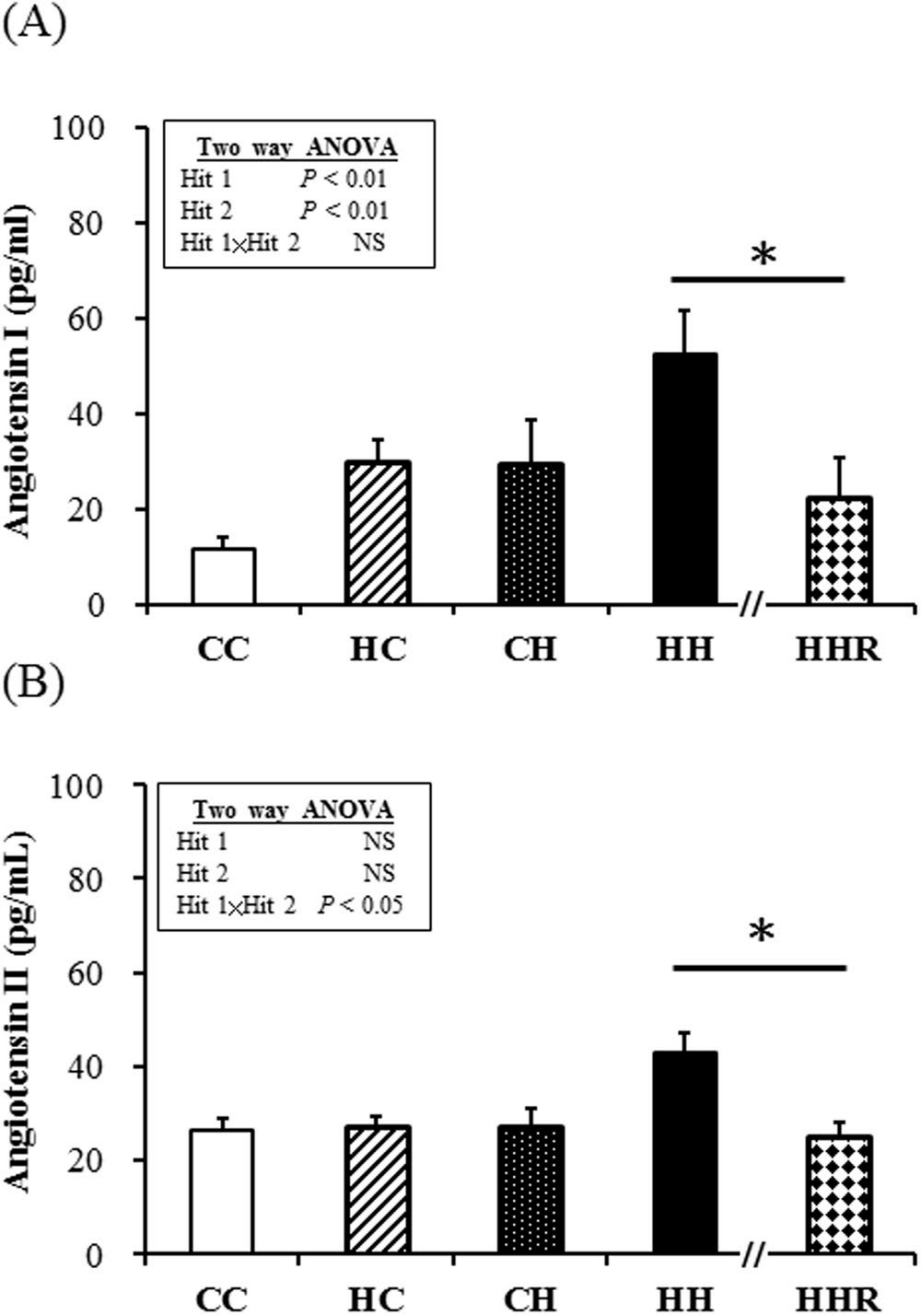
Plasma angiotensin I and II levels. (**A**) Angiotensin I level (**B**) Angiotensin II level. Two-way ANOVA was used to assess the statistical significance of differences among groups and the therapeutic effect of resveratrol was evaluated by Mann-Whitney U test. n = 13 per group in (A) and 6–8 per group in (B). *P < 0.05.

The plasma angiotensin I levels were affected (they increased) both by a maternal high-fat diet and postnatal diet. Resveratrol could reverse the increased plasma angiotensin I levels in the HH group. An interaction between maternal high-fat and postnatal high-fat diet, for the 4-m plasma angiotensin II levels, was observed. *Post hoc* analysis showed higher plasma angiotensin II levels in the HH group than in the CH group. These data indicate that maternal obesity/high-fat diet increased the plasma angiotensin II levels, and sensitized offspring to a second hit, i.e., a postnatal high-fat diet. Resveratrol could reverse the increased plasma angiotensin I and II levels in the HH group (Fig. 2).

### SIRT1

**Figure 3:**
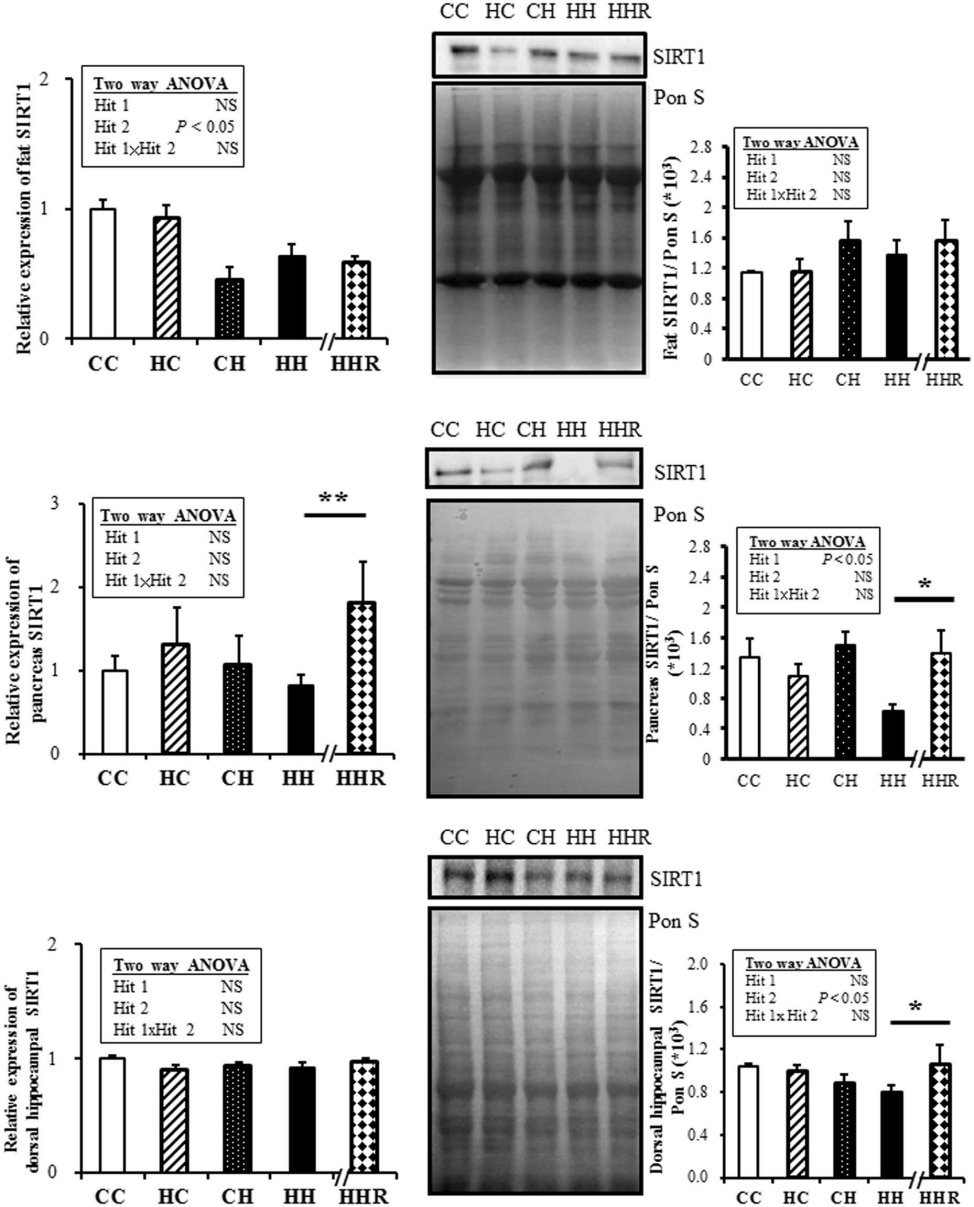
Sirtuin 1 (SIRT1) mRNA expression and abundance. (**A**) fat (**B**) pancreas (**C**) dorsal hippocampus. Two-way ANOVA was used to assess the statistical significance of differences among groups and the therapeutic effect of resveratrol was evaluated by Mann-Whitney U test. Representative immunoblots and densitometric quantification of SIRT-1 are presented. Values are mean ± SEM. Represented full blots are presented in Supplementary Figure S2. n = 11–13 per group in SIRT1 mRNA expression and n = 6–8 in SIRT1 protein amount. There were one outlier in the pancreas and two outliers in the dorsal hippocampus SIRT1 protein abundance, respectively. *P < 0.05; **P < 0.01.

The SIRT1 mRNA expressions of adipose tissue were affected by a postnatal high-fat diet but not by a maternal high-fat diet (Fig. 3). By Western blot (WB), it was found that the fat SIRT1 abundance was not affected either by a maternal high-fat diet or postnatal high-fat diet. Resveratrol did not change the fat SIRT1 mRNA expressions or abundance in the HH group (Fig. 3). The SIRT1 mRNA expressions in the pancreas were not significantly affected by maternal or postnatal high-fat diets. By WB, the SIRT1 expressions of the pancreas were found to be affected by a maternal high-fat diet but not a postnatal high-fat diet. Resveratrol could increase the pancreas SIRT1 mRNA expressions and abundance in the HH group (Fig. 3). We checked the SIRT1 expressions of the dorsal hippocampus because previous studies reported that saturated fat and refined sugar intake was associated with poorer hippocampal-dependent memory function^19^, while the dorsal hippocampus is related to memory processes^20^.We found the SIRT1 mRNA expressions of the dorsal hippocampus were not affected by maternal or postnatal high-fat diets. By WB, the SIRT1 abundance of the dorsal hippocampus was found to be affected (decreased) by a postnatal high-fat diet but not by a maternal high-fat diet. Resveratrol could reverse the decrease in the dorsal hippocampus SIRT1 abundance, in the HH group (Fig. 3).

### RAS

**Figure 4:**
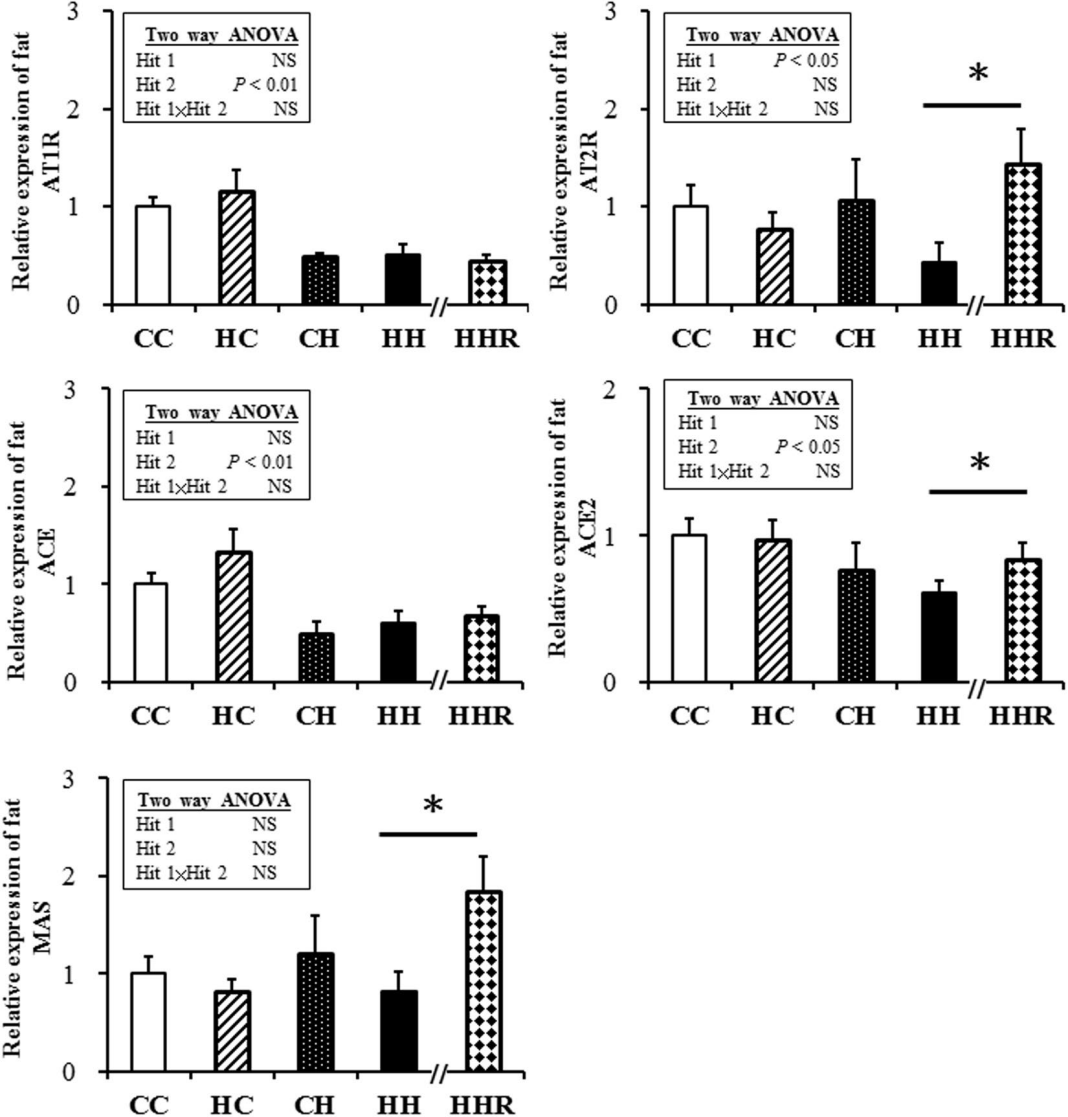
AT1R, AT2R, ACE, ACE2 and MAS in fat. Two-way ANOVA was used to assess the statistical significance of differences among groups and the therapeutic effect of resveratrol was evaluated by Mann-Whitney U test. n = 11–13 per group. *P < 0.05. AT1R, angiotensin II type I receptor; AT2R, angiotensin II type II receptor; ACE, Angiotensin-converting enzyme; ACE2, Angiotensin-converting enzyme 2.

**Figure 5:**
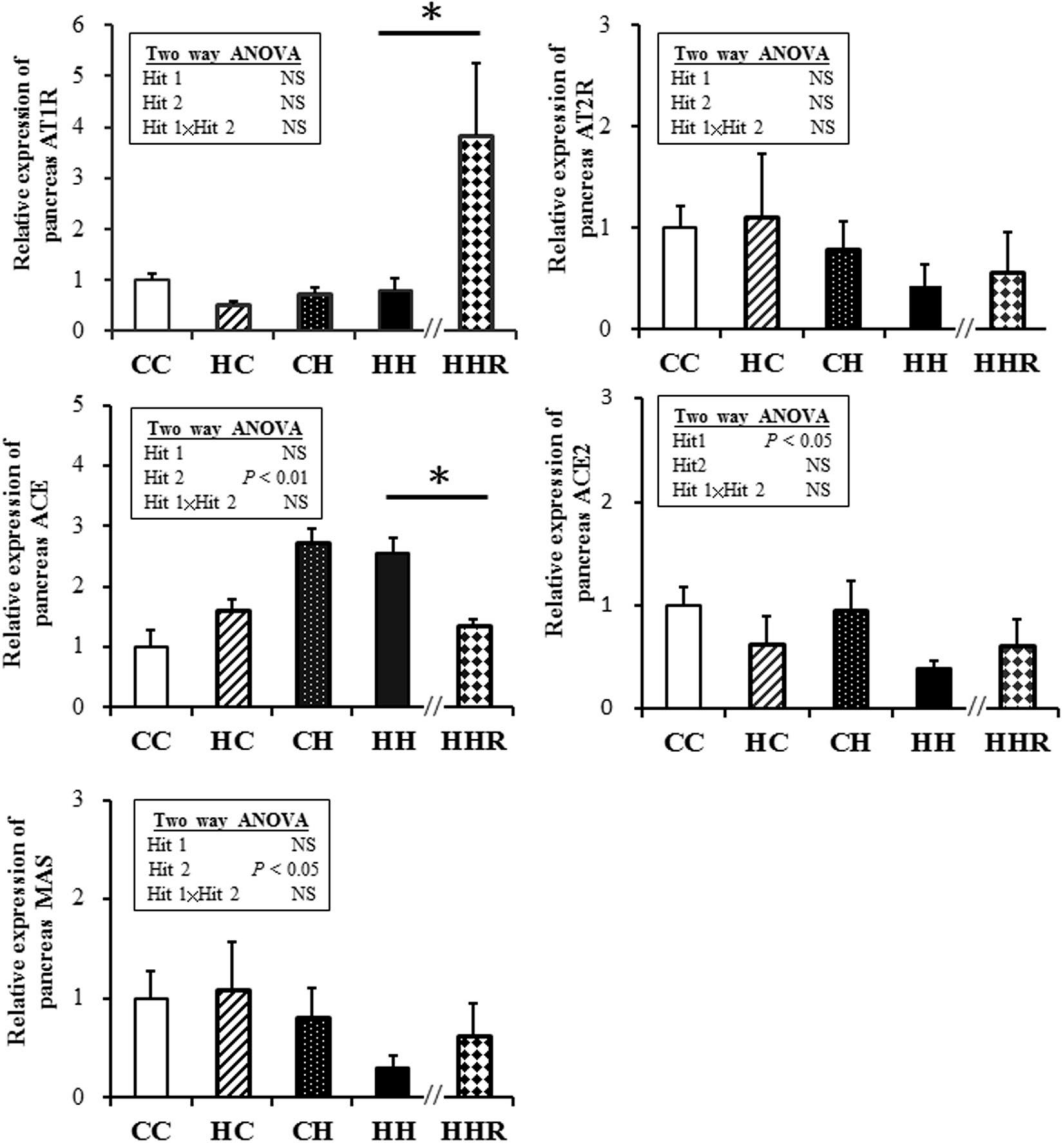
AT1R, AT2R, ACE, ACE2 and MAS in pancreas. Two-way ANOVA was used to assess the statistical significance of differences among groups and the therapeutic effect of resveratrol was evaluated by Mann-Whitney U test. n = 11–13 per group. *P < 0.05.

**Figure 6:**
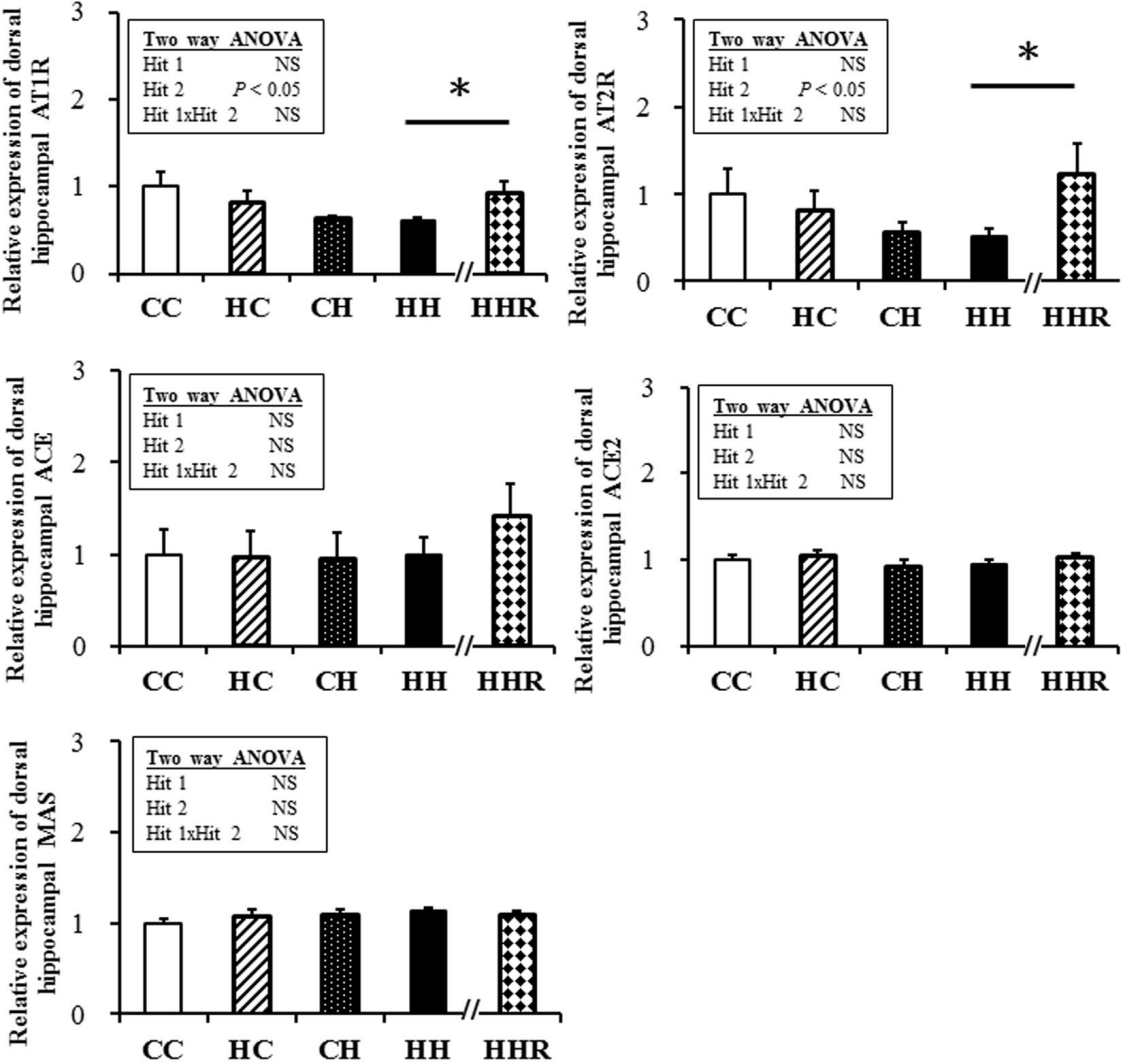
AT1R, AT2R, ACE, ACE2 and MAS in dorsal hippocampus. Two-way ANOVA was used to assess the statistical significance of differences among groups and the therapeutic effect of resveratrol was evaluated by Mann-Whitney U test. n = 11–13 per group. *P < 0.05.

The AT1R, ACE and ACE2 expressions of adipose tissue were affected (decreased) by a postnatal high-fat diet but not by a maternal high-fat diet. The AT2R expressions of the adipose tissue were affected (decreased) by a maternal high-fat diet but not a postnatal high-fat diet. Resveratrol could reverse the decreased AT2R and ACE2 expressions, and increase the MAS expressions in the HH group (Fig. 4). The ACE expressions of the pancreas were affected (increased) by a postnatal high-fat diet but not by a maternal high-fat diet. The ACE2 expressions were affected (but decreased) by a maternal high-fat diet but not by a postnatal high-fat diet. Resveratrol could reverse the increased ACE expression in the HH group (Fig. 5). The AT1R and AT2R expressions of the dorsal hippocampus were both affected (decreased) by a postnatal high-fat diet but not by a maternal high-fat diet. Resveratrol could reverse the expressions of AT1R and AT2R in the HH group (Fig. 6).

## Discussion

We report, here, that maternal obesity/high-fat diet interacts with postnatal high-fat diet to induce features of metabolic syndrome, and resveratrol could alleviate most of the symptoms. Our study showed that: (1) a combination of a maternal high-fat diet and postnatal high-fat diet led to the greatest metabolic disruption; (2) there was an interaction between maternal high-fat and postnatal high-fat diets, in terms of the body weight at 4 months of age, serum leptin level and angiotensin II level; (3) resveratrol could ameliorate the increased body weight, levels of serum ALT, Chol, TG, HDL, leptin, angiotensin I and II, and glucose and insulin AUC values in the HH group; (4) Resveratrol could increase the SIRT1 abundance of the pancreas and dorsal hippocampus in the HH group; (5) the AT2R expressions of fat were decreased through a maternal high-fat diet and the AT2R expressions of the dorsal hippocampus were decreased through a postnatal high-fat diet. Resveratrol could increase the AT2R expression of fat and the dorsal hippocampus in the HH group; (6) the ACE2 expressions of fat were decreased by a postnatal high-fat diet. Resveratrol could increase the ACE2 expression of fat in the HH group; (7) the ACE expressions of the pancreas were increased by a postnatal high-fat diet. Resveratrol could decrease the ACE expression of the pancreas in the HH group.

An increasing number of studies in rodents has demonstrated that offspring-exposure to maternal obesity/overnutrition, during both pregnancy and lactation, leads to a predisposition to a greater increase in adiposity and metabolic dysregulation, than in the case of control dams in which the offspring themselves are challenged with a high-fat diet, after weaning^2,3,7,21–24^. Here, we present a wide spectrum of the metabolic syndrome in rat offspring born to high-fat dams, and having a postnatal high-fat diet. Moreover, we showed that resveratrol can ameliorate most of the features of metabolic syndrome in the HH group. These are consistent with previous reports that resveratrol can improve glucose tolerance and insulin sensitivity^25,26^. Resveratrol is well-known to have a wide variety of effects including anti-oxidant, anti-inflammatory properties and increased mitochondrial biogenesis thereby ameliorates diabetes^27,28^. Interestingly, we also found resveratrol treatment group rats had lower daily calories intake. Therefore, the improvement of blood pressure, leptin, glucose intolerance, etc. may be attributable to appetite suppression related weight loss.

SIRT1 is an important modulator of the maturation and remodeling of adipose tissues^29^. Recently conducted studies also showed that SIRT1 promotes fat mobilization, and stimulates brown remodeling of white fat in white adipose tissue^29^, and the genetic ablation of SIRT1 in adipose tissue leads to increased adiposity and insulin resistance^25^. In addition, a high-fat diet was also reported to trigger the inflammation-induced cleavage of SIRT1 in adipose tissue, to promote metabolic dysfunction^25^. SIRT1 has been also shown to be a positive regulator for pancreatic insulin secretion, which also triggers glucose uptake and utilization. The promotion of insulin secretion was thought to occur through the transcriptional repression of uncoupling protein 2^29^. The activation of SIRT1 by its activators (e.g. resveratrol) may protect against high-fat-induced obesity and insulin resistance^25,27,30^. We previously showed a combination of a maternal and lactation high-fat diet and postnatal high-fat diet resulted in cognition deficit and derangement of the mediators involved in cognition, in the dorsal hippocampus of the adult offspring^31^. Heyward *et al*. showed that mice maintained on a high-fat diet present with impaired hippocampus-dependent spatial memory that may be mediated by the neuroepigenetic dysregulation of SIRT1, within the hippocampus^32^. In our study, the SIRT1 mRNA expression was decreased in the fat tissue by a postnatal high-fat diet and the SIRT1 abundance of the pancreas and dorsal hippocampus were decreased by a maternal high fat diet and postnatal high-fat diet, respectively. The change of SIRT1 mRNA expression was not all the same across groups may be due to different sensitivity to insult among organs. In addition, resveratrol could restore the SIRT1 abundance of the pancreas and dorsal hippocampus. The divergent effect of resveratrol on SIRT1 mRNA expression and protein abundance across the organs may be related with the dose and duration of resveratrol treatment.

White adipose tissue expresses all the critical elements of the RAS, and is a predominant source of circulating angiotensinogen^33,34^. It can generate local angiotensin II to stimulate AT1R and AT2R on adipocytes and other cells within adipose tissue, and contribute to angiotensin II activity on more distant tissues^12^. AT2R is involved in early adipocyte differentiation. AT2R activation can restore normal adipocyte morphology, and improve insulin sensitivity^35^. RAS activation is also closely correlated to both insulin resistance and β cell dysfunction^36^. The underlying mechanism behind this deleterious effect was thought to be related to the negative regulation exerted by angiotensin II through AT1R of several steps of the insulin signaling cascade^37^. The increase in the ACE2/Ang (1–7)/Mas receptor axis could be associated with diminished insulin resistance, through the induction of the activation of insulin-signaling pathways and counteraction of the inhibitory effects of ACE/Ang II/AT1R^38^. The central nervous system (CNS) plays an integral role in maintaining this balance, as it receives and integrates peripheral signals regarding the status of the energy stores and responds to these signals^39–41^. Numerous neuropeptides and other factors influence energy balance by acting directly in the CNS, and accumulating evidence implicates the RAS in this process^12,40^.

In our study, the AT2R expressions were decreased in the fat tissue by maternal high fat diet. The ACE expression of the pancreas was increased by a postnatal high-fat diet and the AT2R expression was decreased in dorsal hippocampus by a postnatal high-fat diet. In addition, resveratrol could reverse them which suggested that resveratrol may ameliorate high-fat diet-induced glycemic dysregulation through regulating ACE/ACE2/AT2R axis.

Oliveira Andrade *et al*. reported that high-fat feed mice had an interaction between angiotensin-(1–7)/Mas axis and sirtuins in the adipose tissue^42^. Clarke *et al*. found that SIRT1 was up-regulated after 5-aminoimidazole-4-carboxamide ribonucleotide (AICAR) treatment, but, conversely, was down-regulated after IL-1β treatment. Chromatin immunoprecipitation analysis demonstrated that SIRT1 was bound to the ACE2 promoter, and that while the binding increased after AICAR treatment, it decreased after IL-1β treatment^43^. In our study, the AT2R and ACE2 expressions were corrected by resveratrol, and thus further explained the close relationship between SIRT1 and RAS.

Many studies have reported the important role angiotensin II plays in connecting insulin resistance and the RAS^44^. Richey *et al*. indicated that an intravenous infusion of angiotensin II induced insulin resistance^45^ and Furuhashi *et al*. reported that the blockade of the RAS improved insulin sensitivity^46^. Moreover, Santos and colleagues reported that Mas-knockout mice presented with dyslipidemia, as well as increased levels of insulin and leptin, and that Mas deletion led to glucose intolerance and reduced insulin sensitivity^47^. In our study, we found that the plasma angiotensin I and angiotensin II levels were increased in the 4 m/o offspring of maternal high-fat and postnatal high-fat diets, and that resveratrol can reverse these, which hint resveratrol may improve high fat-diet-induced metabolic syndrome by decreasing the levels of angiotensin I and angiotensin II.

Our study has a few limitations. First, we did not examine different doses or therapeutic durations of resveratrol. Given that gene–diet interactions vary during different developmental windows, it would be interesting to study whether various therapeutic protocols of resveratrol lead to differential protection on maternal or postnatal high fat-induced adult diseases. Moreover, we haven’t ruled out a reduction in food intake with resveratrol contributing the “normalization” of blood pressure, body weight and leptin, etc. In this connection, a pair-feeding group would allow us to assess the effect of resveratrol independent of food intake. Next, we did IPGTT but not oral glucose tolerance test; this will neglect the effect of incretins. In addition, further study is necessary to measure SIRT1 deacetylase activity and other pathways activated by resveratrol e.g. AMPK.

In conclusion, exposure to a maternal high-fat diet and a post-weaning, high-fat diet appears to “poise” offspring to be hyper-responsive to a high-fat diet, thereby promoting the development of the features related to metabolic syndrome. High-fat diets during both periods resulted in supplementary effects on body weight and plasma leptin and angiotensin II levels. Our findings present important points in the understanding of the complexity of the fetal programming process, and might be particularly useful in the search for efficient therapies against malprogramming. Further studies should be performed to clarify multiple-organ crosstalk, and the interaction of pathogenic mediators in the process of nutritional programming.

## Methods

### Animals and experimental design

This study was carried out in accordance with the recommendations of the Guide for the Care and Use of Laboratory Animals of the National Institutes of Health. The Institutional Animal Care and Use Committee of the Kaohsiung Chang Gung Memorial Hospital approved the protocol. Virgin Sprague-Dawley (SD) rats (BioLASCO Taiwan Co., Ltd., Taipei, Taiwan) were housed and maintained in a facility accredited by the Association for Assessment and Accreditation of Laboratory Animal Care International. The rats were housed in standard rat cage at 22 °C, with a relative humidity of 55%, in a 12 h light/12 h dark cycle.

Two months-old female rats were weight-matched and assigned to receive either a normal diet of regular rat chow (control diet; Fwusow Taiwan Co., Ltd., Taichung, Taiwan; (59.7% carbohydrates, 27.5% protein, 12.6% fat by energy, 3.25 kcal/gm) or a high-fat hypercaloric diet (high-fat diet; D12331, Research Diets, Inc., New Brunswick, NJ, USA; 58% fat [hydrogenated coconut oil], 16.4% protein plus high sucrose [25% carbohydrate] by energy, 5.56 kcal/gm) *ad libitum*, for 5 weeks, before mating as well as during gestation and lactation. Body weight and food intake were measured daily.

We elected to study only male offspring because hypertension, an important component of metabolic disease, occur at an earlier age and at a higher rate in males than females^48,49^. The offspring were weaned at 0.75 month of age, and onto either a normal diet or high-fat diet *ad libitum*, from weaning until 4 months of age. Five experimental groups (n = 13–14 per group) were generated: maternal control diet/postnatal rat chow normal fat diet (CC), maternal high-fat diet/postnatal rat chow normal fat diet (HC), maternal control diet/postnatal high-fat diet (CH), and maternal high-fat diet/postnatal high-fat diet (HH); in addition, a therapeutic group, with resveratrol in drinking water from 2 to 4 months of age, on a maternal high-fat diet/postnatal high-fat diet was raised for comparison (HHR). Resveratrol was prepared twice weekly by dissolving the drug (50 mg) in 5.5 ml of 20% cyclodextrin (Sigma-Aldrich, St. Louis, MO, USA). This solution was then diluted with water. The final concentration is 50 mg/L. Water bottles were wrapped with aluminum foil to protect from light^50^. The mean dose of resveratrol ingested was 10 mg/kg/day.

### IPGTT

After an 8-h fast at postnatal days ~114, blood samples were collected at five time points: before injection and at 15, 30, 60, and 120 min after the intraperitoneal (i.p.) injection of glucose (2 g/kg body weight). Plasma glucose levels were immediately measured using the enzymatic (hexokinase) method, with a glucose assay kit. Serum insulin levels were checked using enzyme-linked immunosorbent assay (Crystal Chem Inc., Downers Grove, IL, USA), as previously reported^51^.

### Blood pressure

Blood pressure was determined in conscious 4 months of age male offspring using an indirect tail-cuff method (BP-2000; Visitech Systems, Inc., Apex, NC, USA) after being systematically trained as previously described^52^.

### Tissue collection and blood sampling

At the age of 4 months, animals were weighed, and then euthanized under isoflurane, by cervical dislocation. The retroperitoneal adipose tissue, pancreas and brain’s dorsal hippocampus were collected. Enzyme-linked immunosorbent assays for the plasma, including TG, Chol, HDL AST, ALT, angiotensin I, angiotensin II and leptin, were performed according to the manufacturers’ protocols.

### Real-time PCR analysis

Briefly, RNA was extracted using TRI Reagent (Sigma, St. Louis, MO, USA), treated with DNase I (Ambion, Austin, TX, USA) to remove DNA contamination, and 2 µg was reverse transcribed (SuperScript II RNase H^–^-Reverse Transcriptase, Invitrogen, Bethesda, MD, USA) with random primers (Invitrogen), in a total volume of 40 µl, as we have already published^53^. Control RT reactions were performed by omitting the RT enzyme, and PCR was amplified to ensure that DNA did not contaminate the RNA. Two-step quantitative real-time PCR was conducted using Quantitect SYBR Green PCR Reagents (Qiagen, Valencia, CA, USA), according to the manufacturer’s protocol on a LightCycler® 480 Real-Time PCR System (Roche Diagnostics Ltd., Taipei, Taiwan). Glyceraldehyde 3-phosphate dehydrogenase (GADPH) was used as a reference. (Supplemental Table 1 shows the primer sequences used in real-time PCR. All samples were run in duplicate (2.5 µl of cDNA/well in a 96-well format). For the relative quantification of gene expression, the comparative threshold cycle (Ct) method was employed. The averaged C_T_ was subtracted from the corresponding averaged GADPH value for each sample, resulting in ΔCt. ΔΔCt was achieved by subtracting the average control ΔCt value from the average experimental ΔC_T_. The fold-increase was established by calculating the 2^−ΔΔCT^ for the experimental vs. control samples.

### WB

WB analysis was done as previously described^54^. Total protein extracts from homogenized cultured cells and liver were lysed in ice-cold RIPA buffer with a protease inhibitor cocktail (Roche, Indianapolis, IN, USA). After centrifugation, the protein concentrations in the supernatants were determined by the DC protein assay kit (Bio-Rad, Hercules, CA, USA). The Western blotting technique was performed to quantify the protein density of SIRT1. We used rabbit anti-rat SIRT-1 antibody (Millipore, Billerica, MA, USA), followed by secondary goat anti-mouse antibody. Bands of interest were visualized using enhanced chemiluminescence (ECL) reagents (PerkinElmer, Waltham, MA, USA) and quantified by densitometry (Quantity One Analysis software; Bio-Rad, Hercules, CA, USA) as the integrated optical density after subtraction of background. The integrated optical density was normalized to Ponceau red staining (Pon S) to correct for any variations in total protein loading. The protein abundance was represented as integrated optical density/Pon S.

### Statistical analysis

Results were analyzed using two-way ANOVA (maternal diet x postweaning diet), followed by LSD *post hoc* tests if the interaction was significant in the first four groups. The therapeutic effect of resveratrol was evaluated by Mann-Whitney U test between HH and HHR group. For all the variables measured, outliers which lay 1.5 interquartile ranges (IQRs) below the first quartile or 1.5 IQRs above the third quartile were removed from the analysis. All analyses were performed using Statistical Package for the Social Sciences (SPSS) software. Values were expressed as mean ± SEM. Significance was defined as *P* < 0.05 for all tests.

## Electronic supplementary material

Supplementary Information
