# Supplementary material for: Combined maternal and postnatal high-fat diet leads to metabolic syndrome and is effectively reversed by resveratrol: a multiple-organ study
Source: Sci Rep. 2018 Apr 4;8:5607. doi: 10.1038/s41598-018-24010-0 (PMC5884801; doi:10.1038/s41598-018-24010-0)

**Combined maternal and postnatal high-fat diet leads to metabolic syndrome and is effectively reversed by resveratrol: a multiple-organ study**

Jiunn-Ming Sheen^a^, Hong-Ren Yu^a^, You-Lin Tain^a,b^, Wan-Long Tsai^a^, Mao-Meng Tiao^a^, I-Chun Lin^a^, Ching-Chou, Tsai^c^, Yu-Ju Lin^c^, Li-Tung Huang^a,d^

**Supplemental Table 1.** qPCR Primer sequences

| Gene | Oligonucleotide sequence 5’→ 3’ |
| --- | --- |
| SIRT1 | 5’ TGGAGCAGGTTGCAGGAATCCA 3’ TGGCTTCATGATGGCAAGTGGC |
| AT1R | 5’ ACCAGGTCAAGTGGATTTCG 3’ ATCACCACCAAGCTGTTTCC |
| AT2R | 5’ CAATCTGGCTGTGGCTGACTT 3’ TGCACATCACAGGTCCAAAGA |
| ACE | 5’ CACCGGCAAGGTCTGCTT 3’ CTTGGCATAGTTTCGTGAGGAA |
| ACE2 | 5’ GCCAGGAGATGACCGGAAA 3’ CTGAAGTCTCCATGTCCCAGATC |
| MAS | 5’ CATCTCTCCTCTCGGCTTTGTG 3’ CCTCATCCGGAAGCAAAGG |
| GAPDH | 5’ TAAAGAACAGGCTCTTAGCACA 3’ AGTCTTGGAAATGGATTGTCTC |

SIRT1, sirtuin-1; AT1R, angiotensin II type I receptor; AT2R, angiotensin II type II receptor; ACE, angiotensin-converting enzyme; ACE2, angiotensin-converting enzyme 2; GAPDH, glyceraldehyde 3-phosphate dehydrogenase.

**Legends**

**Supplemental Figure. 1 Maternal body weight and offspring body weight.** (A) Female rats were fed with a chow diet (n = 12) or a high-fat diet (n = 13) and weighed weekly. High-fat diet group was heavier than the control group from 1 week after taking different diet and thereafter (B) Male baby weights on the second day of birth (n = 27 in the control group, 42 in the high-fat diet group). The body weight born by mother taking high-fat diet was lower. Two groups were evaluated by Mann-Whitney U test. * *P* < 0.05. HFD, high-fat diet.

**Supplemental Figure. 2 Full blots of Fig. 3 in the manuscript. The represented figure is indicated.**

**Supplemental Figure. 1**


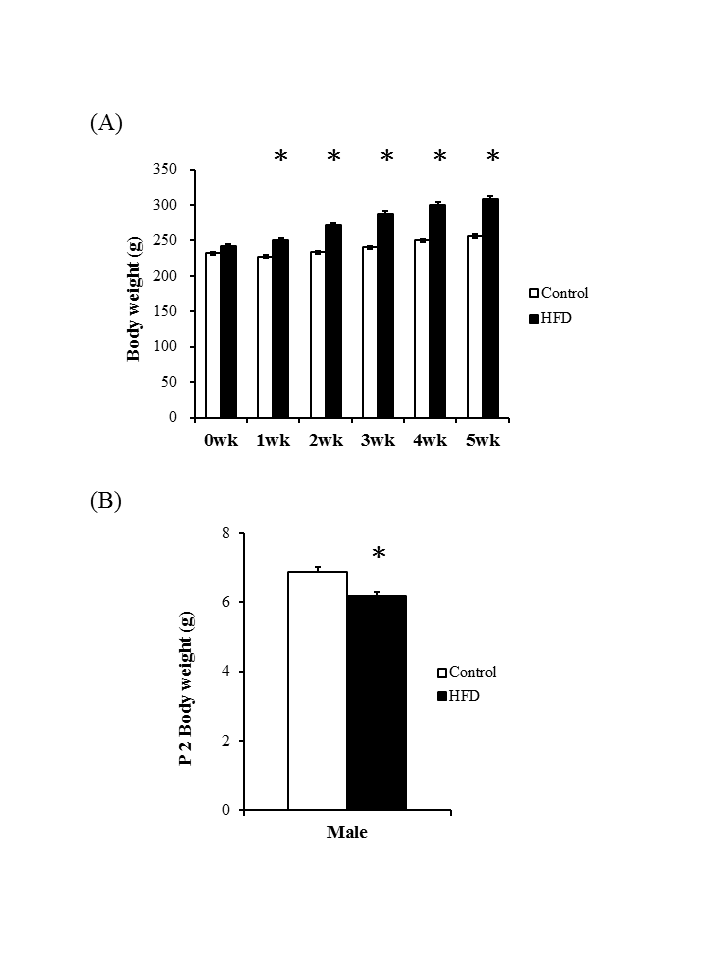


**Supplemental Figure. 2**


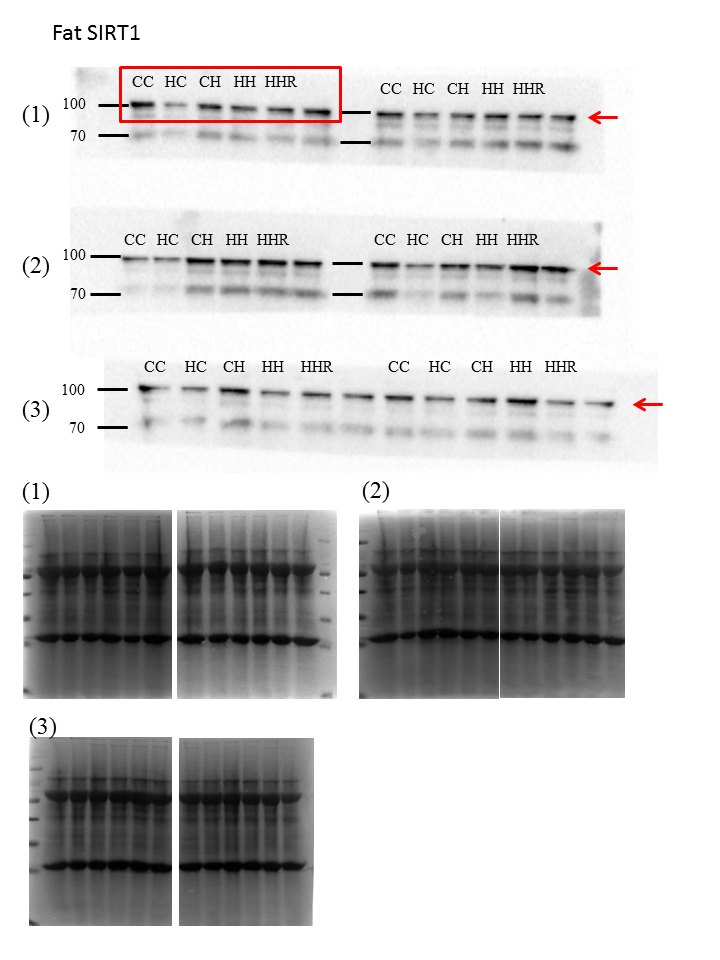


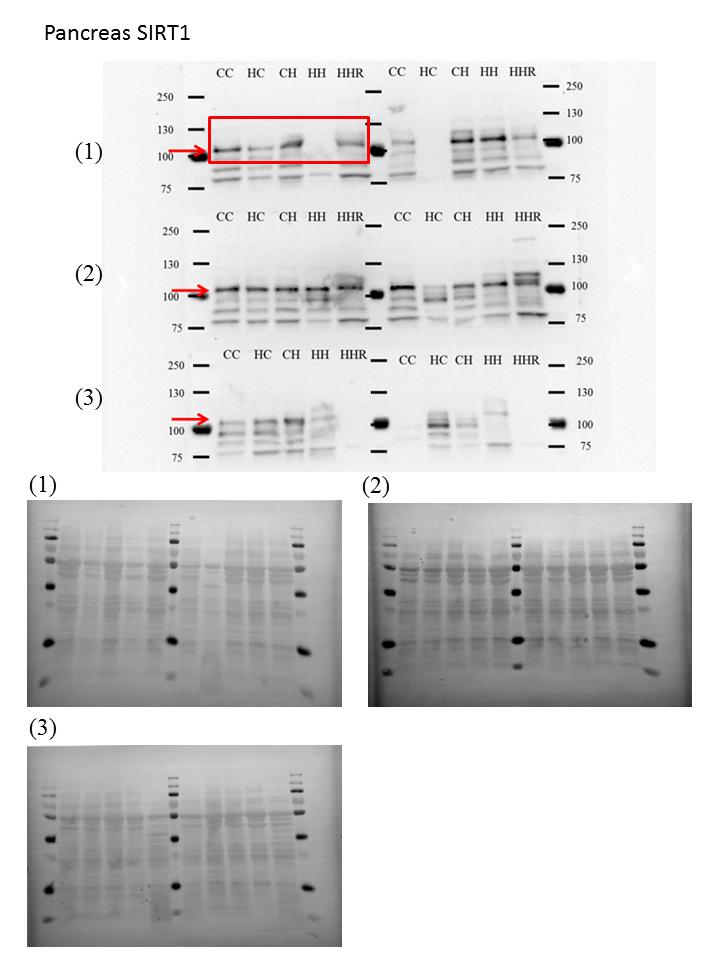


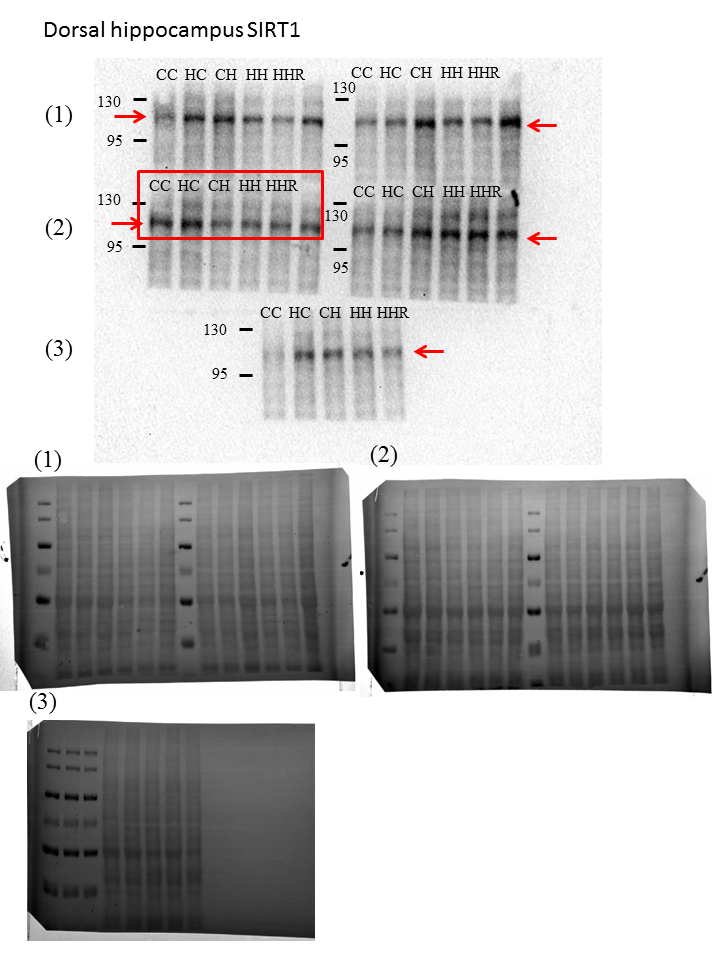

Supplement: Supplementary file 1 — Supplementary Information [file 41598_2018_24010_MOESM1_ESM.doc]
